# Changes in smoking prevalence after the enforcement of smoking control regulations in urban Shanghai, China: Findings from two cross-sectional surveys

**DOI:** 10.18332/tid/91095

**Published:** 2018-06-01

**Authors:** Xiaolin Qian, Haiyan Gu, Lan Wang, Xian Wang, Zeliang Xuan, Pinpin Zheng, Chaowei Fu

**Affiliations:** 1 Department of Chronic Disease Prevention and Control, Xuhui District Center for Disease Control and Prevention, Shanghai, China; 2 School of Public Health, Fudan University, Shanghai, China

**Keywords:** tobacco control, smoking prevalence, smoke-free policy, smoking ban

## Abstract

**INTRODUCTION:**

The Smoking Control Regulation in Public Places (hereafter, the ‘regulations’) has been implemented in Shanghai since 2010. This study explores the changes in smoking prevalence and its influencing factors among urban Shanghai residents.

**METHODS:**

Two rounds of household investigations (the Health Status and Health Service Utilization Survey) were carried out using a multistage probability proportionate-to-size sampling method in an urbanized district in 2010 and 2015. Descriptive and logistic regression analyses were applied to the statistics.

**RESULTS:**

From 2010 to 2015, the standardized current smoking rate fell from 24.8% to 19.1% (38.3% to 32.0% among men, and 1.9% to 1.4% among women). Meanwhile, the standardized smoking cessation rate increased from 18.1% to 23.3%. Smoking prevalence in respondents aged 45 to 59 years was still higher than that of other age groups. Changes in smoking prevalence and cessation rates were more obvious in respondents aged 30–44 and over 75 years. Sex, age, education, marital status, and alcohol use were influencing factors of current smoking, while sex, age and alcohol use were influencing factors of smoking cessation.

**CONCLUSIONS:**

The implementation of smoking control regulations may be beneficial for reducing smoking and increasing smoking cessation, especially among middle-aged and older men. Nevertheless, tobacco control in urban Shanghai still faces huge challenges. Therefore, more targeted and comprehensive measures should be taken.

## INTRODUCTION

Tobacco use is one of the most serious public health problems in the world today. The annual deaths caused by cigarette smoking globally are as high as 6 million^1^. China is the largest tobacco producer and consumer in the world, with more than 300 million smokers, while 740 million non-smokers suffer from the dangers of secondhand smoke (SHS). More than 1 million deaths are caused by smoking-related diseases annually^2^. Within the last decade, more provincial and city administrations in China have enacted policies to control tobacco use and SHS.

In the absence of national smoke-free laws, in March 2010 Shanghai became the first city in China to issue provincial-level legislation to limit indoor smoking in some public places within the city. Although this legislation is a partial smoking ban that only prohibits smoking in 13 types of public places, including kindergartens, schools, hospitals, stadiums, public service places, shopping malls, libraries, theaters, and museums, it is nevertheless a big step. Tobacco-control measures included posting no-smoking signs, removing smoking settings, and discouraging smokers. Clear evidence shows that legislative smoking bans do lead to improved health outcomes through reduction in SHS, such as reducing admissions for acute coronary syndrome^3-5^. This legislation is known to be helpful in creating a smoke-free environment, but little is known about the effects of the implementation of the legislation in Shanghai. We compared the changes in smoking status from the first and sixth years of the enforcement of the legislation and explored the influencing factors to provide evidence for future tobacco control strategies.

## METHODS

### Study area

The study was carried out among residents in the Xuhui District of Shanghai, China, which is located southwest of Shanghai’s city center. According to the Statistical Communiqué of the People’s Republic of China on the 2015 National Economic and Social Development, the district covers 54.76 square kilometers and has a permanent resident population of 1.085 million people^6^. Xuhui serves as a western gateway to Shanghai’s suburban districts and other neighboring cities.

### Participants and sampling strategies

The data in this study were obtained from Shanghai residents’ responses to the Health Status and Health Service Utilization Survey during two monitoring studies performed from October to November in both 2010 and 2015. The first study was performed 7 months after the implementation of the Smoking Control Regulation in Public Places (hereafter, the ‘regulations’). The two monitoring studies applied the same sampling method, i.e. a multistage probability proportionate-to-size sampling method. In the first phase, a total of 13 communities were selected randomly in Xuhui District. In the second phase, 100 neighborhood committees were selected randomly according to each community’s population size. In the third phase, a starting point was randomly selected to determine the first household to investigate from about 45 to 55 families in each neighborhood committee.

Residents aged 15 years and older who lived in the community for at least 6 months were eligible for the survey. A total of 11 347 residents in 2010 and 10 388 residents in 2015 completed the survey with efficiency rates of 94.1% and 93.0%, respectively. The overall response rates were 90% and 91% in 2010 and 2015, respectively. To avoid a cluster effect, the study selected one resident in each family whose birthday was nearest to the investigation date. Therefore, the subsamples included in this study were 4986 and 4955 in 2010 and 2015, respectively.

### Measurement and instrument

The survey included sociodemographic characteristics and smoking-associated questions, among others. The sociodemographic data included the respondents’ age, marital status, ethnicity, education, occupation, and income. The measure of tobacco-smoking prevalence was based on questions adapted from the Global Adult Tobacco Survey (GATS)^7^. Apart from past and present smoking status, smoking frequency and number of tobacco products per day were also monitored. (Questions: have you ever smoked; how old did you start to smoke; by the end of the survey day, have you ever smoked 100 cigarettes; how often is the current frequency of smoking; how many cigarettes did you smoke per day in the last year?)

Smoking rate refers to the proportion of people who had ever smoked 100 cigarettes, while current smoking rate refers to the proportion of people who had ever smoked 100 cigarettes and are still smoking during the survey, and smoking cessation rate refers to the proportion of smokers who quit smoking during the survey.

### Data collection procedures

Data were collected face-to-face by trained family physicians and public health personnel. If the selected family refused to participate in the survey or no one was present at three visits, the interviewers would skip to the next family. In terms of quality control, the School of Public Health at Fudan University randomly conducted a secondary telephone survey on 10% of the participant families to ask eight important questions. If three or more questions were inconsistent with the questionnaire responses, the questionnaire was deemed not reliable and was returned for reinvestigation (did not qualify). The qualification rates were 95.6% and 98.1% in 2010 and 2015, respectively.

### Statistical analysis

Data analyses were performed using SPSS software (version 16.0; SPSS, Chicago, IL, USA). Sample weights were used in the data analyses. The sampling weight of each stage was the reciprocal of the sampling probability of the stage. The total weight of the sampling was the product of the weight of each stage. Descriptive statistics were used to estimate smoking prevalence. We performed Student’s t-tests when response values were continuous and chi-squared tests when values were categorical. Multiple logistic regression was used to determine the influencing factors on current smoking and smoking cessation. Current smoking and smoking cessation were considered dependent variables. Statistically significant smoking-associated factors in univariate analysis were included in the multiple logistic regression model. A p-value less than 0.05 was considered statistically significant. To eliminate the influence of age, we adjusted ages based on the sixth census of the Shanghai population^6^.

## RESULTS

### Demographic characteristics

In 2010, the average age of the 4986 participants was 58.4±16.9 years while in 2015 the average age of the 4955 participants was 59.6±16.2 years. Significant differences in smoking were observed for most of the demographic characteristics except for marital status (Table 1).

**Table 1:** Comparison of demographic characteristics among participants in 2010 and 2015 [Number (%)]

*Characteristics*	*2010 (n=4986)*	*2015 (n=4955)*	*χ^2^*	*p*
**Sex**				
Male	3125 (62.7)	2863 (57.8)	24.87	0.00
Female	1861 (37.3)	2092 (42.2)		
**Age groups (yrs)**				
15–29	369 (7.4)	256 (5.2)	124.53	0.00
30–44	676 (13.6)	736 (14.9)		
45–59	1656 (33.2)	1376 (27.8)		
60–74	1273 (25.5)	1715 (34.6)		
≥75	1012 (20.3)	872 (17.6)		
**Education level**				
Primary school or less	570 (11.4)	423 (8.6)	26.62	0.00
Secondary school	1207 (24.2)	1211 (24.4)		
High school graduate	1496 (30.0)	1590 (32.1)		
College or above	1713 (34.4)	1730 (34.9)		
**Marital status**				
Single	413 (8.3)	385 (7.8)	2.01	0.57
Married	3738 (75.0)	3702 (74.7)		
Divorced/widowed	822 (16.5)	860 (17.4)		
Other	13 (0.2)	8 (0.1)		

### Smoking status

In 2010, the average starting age for smoking was 23.1±7.3 years (male, 22.9±7.1 years; female, 29.2±12.4 years), while the average starting age for smoking in 2015 was 22.1±6.6 years (male, 22.0±6.5 years; female, 25.0±8.7 years). This indicates that the average starting age for smoking decreased from 2010 to 2015 (p<0.01). As shown in Table 2, the standardized overall current smoking rate decreased from 24.8% in 2010 to 19.1% in 2015 (38.3% to 32.0% among men, and 1.9% to 1.4% among women). Men aged between 45–59 years had the highest smoking and current smoking rates. There was a statistically significant decrease in male smoking rates and current smoking rates from 2010 to 2015 among respondents aged 30–59 (p<0.01) and over 75 years old (p<0.05). The standardized smoking cessation rate increased from 18.1% in 2010 to 23.3% in 2015 (17.7% to 22.7% among men, and 28.1% to 40.0% among women). Changes in cessation rates from 2010 to 2015 were more obvious among men aged 30–44 (p<0.01) and over 75 years (p<0.05). The average daily tobacco consumption was lower in 2015 than in 2010 (14.3±9.0 vs 15.3±28.2 cigarettes); however, no significant difference was detected (p>0.05).

**Table 2:** Smoking status of different age groups in 2010 and 2015

*Year*	*Age (yrs)*	*Smoking rate (%)*			*Current smoking rate (%)*			*Smoking cessation rate (%)*		
		*Male*	*Female*	*Total*	*Male*	*Female*	*Total*	*Male*	*Female*	*Total*
2010	15–	29.5	4.8	19.8	29.0	4.1	19.2	6.1	28.6	8.2
	30–	47.4	3.3	31.7	46.0	2.9	30.6	7.8	12.5	7.9
	45–	67.6	2.3	45.7	62.6	2.2	42.3	9.5	15.4	9.6
	60–	45.6	1.3	29.6	33.6	1.1	21.8	29.9	50.0	30.2
	75–	33.6	3.3	19.9	15.7	1.7	9.4	55.9	46.7	55.2
	Total	50.3	2.6	32.5	42.0	2.0	27.1	19.5	30.6	19.8
	Standardized rate	45.8	2.4	29.7	38.3	1.9	24.8	17.7	28.1	18.1
2015	15–	28.7	1.6	15.2	28.7	1.6	15.2	2.7	0.0	2.6
	30–	30.6**	2.5	18.3**	28.0**	1.6	16.4**	18.9**	50.0	20.7**
	45–	57.2**	2.5	35.1**	53.8**	2.7	33.1**	10.0	35.7	10.8
	60–	46.2	1.4	28.8	34.7	1.2	21.7	30.5	44.4	30.8
	75–	28.2*	1.4	15.3**	10.4**	0.7	5.7**	70.9*	66.7	70.7*
	Total	43.5**	1.9	25.9**	35.1**	1.6	20.9**	24.9**	43.6	25.5**
	Standardized rate	39.6	1.7	23.7	32.0	1.4	19.1	22.7	40.0	23.3

** Comparing the results in 2015 with those in 2010. Chi-squared p<0.01

* p<0.05.

### Current smoking-associated influencing factors

A multivariable logistic regression analysis (Table 3) showed that in addition to income, sex, age, education, and alcohol consumption, were significantly associated with current smoking in the both surveys. Men were more likely to be currently smoking than women. Residents aged 45 to 59 years were more likely to be current smokers than other age groups. Education level was a protective factor for current smokers (adjusted odds ratio, AOR=0.77 in 2015). Heavy drinkers were more likely to be current smokers. Income showed a significant influence on current smoking status in 2015 (AOR=0.88), i.e. with an increase in income by month per capita people were less likely to be current smokers.

**Table 3:** Current smoking-associated influencing factors in 2010 and 2015

*Influencing factors*	*2010*				*2015*			
	*OR*	*p*	*AOR*	*p*	*OR*	*p*	*AOR*	*p*
**Sex** (Male=0, Female=1)	0.03	0.00	0.03	0.00	0.03	0.00	0.03	0.00
**Age groups** (yrs) (15–29=0)		0.00		0.00		0.00		0.00
30–44	1.86	0.00	1.41	0.08	1.10	0.65	0.78	0.29
45–59	3.08	0.00	1.93	0.00	2.76	0.00	1.47	0.09
60–74	1.17	0.28	0.64	0.03	1.54	0.02	0.57	0.02
≥75	0.44	0.00	0.20	0.00	0.34	0.00	0.12	0.00
**Marital status** (Married=0)		0.00		0.08		0.00		0.41
Single	0.59	0.00	0.81	0.21	0.51	0.00	0.83	0.29
Divorced/widowed	0.24	0.00	1.36	0.07	0.59	0.00	1.20	0.27
**Education level**	0.94	0.01	0.76	0.00	0.91	0.00	0.77	0.00
**Alcohol consumption**								
(Never or occasionally=0)		0.00		0.00		0.00		0.00
1–2 times weekly	5.64	0.00	3.12	0.00	5.28	0.00	2.96	0.00
≥3 times weekly	7.01	0.00	3.51	0.00	10.15	0.00	4.88	0.00
**Income by month per capita**								
(RMB) (<2000=0)	0.90	0.00	0.96	0.18	0.82	0.00	0.88	0.00

AOR: adjusted odds ratio

### Smoking cessation-associated influencing factors

The multivariable logistic regression analysis (Table 4) showed that sex, age and alcohol consumption were significantly associated with smoking cessation in 2015. Women had a stronger intention to quit smoking (AOR=2.22).As smokers age they tend to quit smoking; however, smokers who consume alcohol, three or more times weekly, find it more difficult to give up smoking.

**Table 4:** Smoking cessation-associated influencing factors in 2010 and 2015

*Influencing factors*	*2010*				*2015*			
	*OR*	*p*	*AOR*	*p*	*OR*	*p*	*AOR*	*p*
**Sex** (Male=0, Female=1)	2.02	0.01	1.59	0.19	2.01	0.02	2.22	0.01
**Age groups** (yrs) (15–29=0)		0.00		0.00		0.00		0.00
30–44	0.69	0.38	0.92	0.87	9.35	0.03	9.62	0.03
45–59	0.89	0.75	1.35	0.49	5.05	0.11	6.06	0.08
60–74	3.43	0.00	5.35	0.00	16.25	0.01	19.65	0.00
≥75	9.72	0.00	15.69	0.00	74.88	0.00	98.19	0.00
**Marital status** (Married=0)		0.00		0.26		0.01		0.63
Single	0.90	0.69	1.73	0.09	0.55	0.06	0.94	0.87
Divorced/widowed	2.17	0.00	0.79	0.35	1.67	0.01	0.72	0.19
**Education level**	0.81	0.00	0.99	0.85	0.88	0.06	1.03	0.73
**Alcohol consumption**								
(Never or occasionally=0)		0.00		0.00		0.00		0.00
1–2 times weekly	0.48	0.00	0.63	0.10	0.64	0.06	0.63	0.08
≥3 times weekly	0.48	0.00	0.51	0.00	0.42	0.00	0.42	0.00

AOR: adjusted odds ratio

## DISCUSSION

This study evaluated the change in smoking prevalence after the enforcement of smoking control legislation in Shanghai. After the enforcement of the partial smoke-free legislation for 5 years, the age-standardized current smoking rate in urban Shanghai declined from 24.8% to 19.1% (38.3% to 32.0% in men, 1.9% to 1.4% in women) while the national current smoking rate (GATS) changed from 28.1% in 2010^2^ to 27.7% in 2015^8^. This reduction achieved statistical significance among men aged 30–59 years and aged 75 years and older. The smoking cessation rate increased from 18.1% to 23.3%, increasing as individuals aged, and showed a significant change in the male groups aged 30–44 years and 75 years and older.

The national smoking control legislations have not been implemented in China. Only two official national documents related to smoking control have been published to date, which focus on medical practitioners and adolescents, respectively. In 2010, Shanghai seized the opportunity to host the World Expo, which effectively advanced the tobacco control legislation process. Although the regulations enforced in March 2010 were not comprehensive, they followed the eighth article of the Framework Convention on Tobacco Control (World Health Organization), i.e. ‘Protection from exposure to tobacco smoke’, to clearly define smoke-free public places and confirm the smoke-free units’ duties and legal responsibilities.

According to a 2016 Cochrane review, the evidence is inconsistent with the impact of legislative bans on smoking prevalence and tobacco consumption3. The decline in smoking rates after the smoke-free legislation found in this study was similar to the domestic studies done in Beijing^9^ and Guangzhou10, and foreign studies done in Canada^11^, Spain^12^ and the US^13^. In studies using an interrupted time series design in States of the USA or Canadian provinces that have implemented comprehensive smoke-free legislation, an immediate decline in smoking prevalence was observed in some jurisdictions14. In addition, studies using uncontrolled before-and-after methods reported significant reductions in smoking prevalence after the introduction of smoking bans in 14 of the 17 States of the USA in 2012^15^. However, comprehensive smoke-free legislation may have a greater influence than partial smoke-free legislation^16,17^, with more dramatic declines within less time. For example, the smoking prevalence in Saskatoon, Canada^11^ dropped 5.9% within 1 year after the ban.

In our study, a multivariate analysis of current smoking showed that sex, age, marital status, educational level, alcohol consumption and income could be associated with current smoking behavior. The distinct difference between male and female arose from traditional Chinese culture influences. The risks of smoking for men aged 45–59 years, divorced or widowed, with lower education and income level, and drinking group, were relatively higher. These influencing factors were consistent with findings reported previously^18-20^. Middle-aged men were heavily affected, which was expected because this group has stronger social activities, while the legislation reduces the social acceptability of smoking and restricts their opportunities for smoking. Divorced or widowed people may have emotional distress that likely leads them to smoke for comfort. The findings were also compatible with smoking cessation-associated factors. The higher smoking cessation rate among the oldest group was likely due to their declining health, which stimulated their self-health protection consciousness and their determination to quit smoking. Although it is never too late to quit smoking, whether smoking cessation was due to illness instead of an improvement in health education is a big question. Self-reported alcohol consumption risk was positively associated with smoking; therefore, the interaction between alcohol consumption and smoking deserves further study.

In addition, no significant drop in smoking rates of respondents aged 15 to 29 years was observed, although the overall smoking rate dropped. Furthermore, the smoking cessation rates of respondents aged 15 to 29 years remained low. This was consistent with China’s domestic studies^8^, which suggest that the current partial smoke-free policy was inadequate in reducing smoking prevalence and affecting smokers’ behavior. The young tend to underestimate the dangers of tobacco and the possibility of addiction. The younger the adolescent starts smoking, the greater the likelihood of becoming a regular user, and the less likely he or she will be to give up smoking^21^. The tobacco industry has adopted a range of strategies aimed at young people. Although the existing advertising law explicitly forbids traditional tobacco advertisements, the tobacco industry uses new media for its marketing campaigns, such as websites and social media. Because of the rapid, extensive and reproducible characteristics of new media, this kind of tobacco marketing is particularly harmful to young people^22^.

The WHO established MPOWER, which packages the six most important and effective strategies on tobacco control. MPOWER suggests: Monitoring tobacco use; Protecting people from tobacco smoke; Offering help to quit tobacco use; Warning about the dangers of tobacco; Enforcing bans on tobacco advertising, promotion and sponsorship; and Raising tobacco taxes. The Netherlands’ SimSmoke tobacco control policy stimulation model showed that smoking prevalence decreased by as much as 21% in the first year, then by 35% in the next 20 years by following a set of policies recommended by MPOWER^17^.

Shanghai had a good start in implementing the smoke-free legislation, which represents the P (i.e. Protecting people from tobacco smoke) in MPOWER. During these 5 years, Shanghai not only promoted a smoke-free environment in schools, hospitals, enterprises and various legal non-smoking places, but also mobilized government employees, teachers and medical staff to play an exemplary role in tobacco control activities and to constantly improve public awareness of tobacco control. Law enforcement departments focused on increasing the frequency of law enforcement checks and administrative penalties for key public spaces, such as Government agencies, Internet cafes, entertainment, catering, and bus stations, to enhance the overall legal enforcement. Meanwhile, the government integrated smoke control into the construction of national demonstration area for comprehensive prevention and control of noncommunicable diseases. Xuhui District in Shanghai was among the first in China to establish such a national demonstration area in 2011. In addition to these measures, tobacco use and relevant health outcomes were monitored. In May 2012, a service network for smoking cessation was launched, which consisted of short smoking cessation interventions, smoking cessation outpatient services, and a smoking cessation hotline. Many other aspects of the service network certainly still need to be improved, e.g. health warnings are not large, bold or graphic, and cigarette taxes are low. Comprehensive measures in individual and population-based interventions may play a role in tobacco control. The decline in smoking rate could be associated with socioeconomic development, improvement in general education, and in particular health education, and increased activities against tobacco use. The interaction of internal and external factors can lead to changes in smoking behavior.

Our study is a large population-based survey of the changes in the prevalence of smoking and its influencing factors in urban Shanghai. However, this study has its limitations. The data used to estimate the impact of the legislation are limited and some important indicators, such as SHS exposure, awareness and knowledge of the regulations, and behavioral changes were not included in the study. The collection of information about the effect of smoking fines to dissuade smoking, and on smoking cessation outpatient services, was limited.

## CONCLUSIONS

We observed a decrease in smoking rates after the implementation of the partial smoke-free legislation, especially among middle-aged and older men. While we cannot conclude that this decrease arose because of the regulations, it is nevertheless plausible that the regulations contributed to this decrease. Nevertheless, men aged 45 to 59 years are still the main target of tobacco control interventions in urban Shanghai. More attention on adolescent and youth smoking is also needed. However, tobacco control in urban Shanghai still faces many challenges and a comprehensive tobacco control strategy is urgently needed.
